# Family dysfunction, parenting stress, and child mental health: associations with bullying involvement and the moderating role of neighborhood support

**DOI:** 10.3389/fpsyg.2025.1644696

**Published:** 2025-09-04

**Authors:** Qianyu Zhou, Chaohui Lin, Xiang Guo

**Affiliations:** ^1^School of Law, Wuhan University, Wuhan, China; ^2^Faculty of Law, University of Macau, Taipa, Macao SAR, China

**Keywords:** family dysfunction, bully involvement, parental aggravation, neighborhood support, children’s mental health

## Abstract

**Introduction:**

Childhood bullying is widespread and closely tied to family stress and mental health problems. When family relationships are strained and parents experience high stress, children’s psychological well-being may erode, increasing their chances of bullying involvement as aggressors or victims. This study assessed (1) whether family dysfunction (FD) affects bullying through a sequential pathway of parental aggravation (PA)—a marker of parenting stress—and children’s mental health (CMH), and (2) whether perceived neighborhood support (NS) tempers this chain.

**Methods:**

We used data from the 2020–2023 National Survey of Children’s Health, a pooled cross-sectional, nationally representative sample totaling roughly 114,639 U. S. children aged 6–17 years. Primary measures were a composite FD index, a four-item PA scale, parent-reported CMH difficulties, perceived NS, and past-year bullying involvement. A moderated serial-mediation model tested the indirect pathway from FD to BI via parenting stress and CMH, with NS specified as a moderator.

**Results:**

Higher levels of FD predicted greater PA and poorer CMH. Both parenting stress and child mental health difficulties were, in turn, associated with higher odds of BI. Serial mediation analysis supported a significant indirect effect of FD on BI through parenting stress and CMH. This mediated pathway was significantly weaker at high NS levels, indicating a buffering role.

**Discussion:**

Findings suggest that nurturing neighborhood environments can offset some of the negative consequences of FD and parental stress on children’s involvement in bullying. Prevention initiatives that simultaneously strengthen family functioning and neighborhood cohesion may therefore effectively curb youth bullying.

## 1 Introduction

Bullying involvement among young people, whether as aggressors, targets, or both, constitutes a pervasive public-health challenge with extensive sequelae. U. S. surveillance studies estimate that roughly one-fifth to one-third of children and adolescents either engage in or endure bullying behaviors, underscoring the breadth of the problem (Nansel et al., 2001). Comparable international data indicate that nearly one in three youths worldwide report some form of peer victimization, confirming that bullying is a global phenomenon rather than a uniquely American issue (Otchet, 2018; Thomsen et al., 2024). Importantly, bullying cannot be dismissed as a harmless rite of passage; longitudinal evidence links both perpetration and victimization to elevated risks of depression, anxiety, academic under-achievement, and persistent physical health complaints extending into adulthood. Because these concurrent emotional and behavioral difficulties carry substantial long-term costs for individuals and society alike, preventing bullying remains a pressing public-health imperative. Although most prevention initiatives concentrate on schools and peer dynamics, bullying is a multifaceted behavior embedded in several ecological layers (Sigurdson et al., 2015; Wolke and Lereya, 2015; Ye et al., 2023). Emerging scholarship underscores the family milieu as a pivotal—yet comparatively understudied—determinant of bullying involvement, but the mechanisms by which adverse home conditions translate into peer aggression remain insufficiently mapped (Shao et al., 2024). Developmental frameworks posit that children’s actions are molded not only by personal attributes and school environments but also by familial processes and neighborhood characteristics (Bowes et al., 2009). Bronfenbrenner’s ecological systems theory, for example, situates child development within nested contexts and suggests that family interactions and community conditions have both direct and indirect effects on youth behavioral outcomes.

However, empirical work has only begun to clarify how dysfunction within families feeds into youths’ bullying involvement (Nocentini et al., 2019). Integrated, process-oriented frameworks that track how adverse home conditions trigger bullying through intermediate psychological mechanisms—and identify when community resources blunt these pathways—remain rare (Akat et al., 2025; Grama et al., 2024). To narrow this gap, the present study analyzes National Survey of Children’s Health data from 2020 to 2023, testing a moderated serial-mediation model in which family dysfunction elevates bullying risk via a cascade of parenting stress and child mental-health difficulties, while neighborhood support is posited as a buffering force.

### 1.1 Family dysfunction and youth bullying involvement

Converging evidence links dysfunctional family climates to greater bullying perpetration and victimization. “Risky families,” characterized by chronic conflict, aggression, and emotional coldness, erode children’s emotion-regulation skills and social adjustment (Repetti et al., 2002). In such settings, youth may acquire maladaptive interpersonal scripts or develop vulnerabilities that make them likelier to bully or be bullied by peers (Baldry, 2003). Population-based studies show that exposures such as household dysfunction, inter-parental violence, inconsistent discipline, and general family chaos substantially raise the odds of occupying any bullying role. For instance, children in a Portuguese birth-cohort who experienced domestic violence, parental substance misuse, or mental illness were markedly more likely to be classified as bullies, victims, or bully-victims at age 10 (Fraga et al., 2021). Likewise, a systematic review of 154 studies concluded that contextual risks (e.g., parental psychopathology, family violence) and relational risks (poor parent–child warmth, abuse) reliably predict bullying involvement across roles (Nocentini et al., 2019). Longitudinal work further indicates that, net of baseline behavior problems, early exposure to family adversity forecasts subsequent bully, victim, and bully-victim statuses (de Vries et al., 2018).

Recent mechanistic work illuminates the pathways linking family dysfunction to bullying. A three-wave study of 1,060 families showed that chronic inter-parental conflict increased parenting stress, which eroded parental psychological flexibility; this sequential pathway predicted rises in adolescents’ externalizing and relational aggression 2 years later (Wang et al., 2025). Complementing this process view, a prospective Dutch cohort demonstrated that fathers’ prenatal distress, fathers’ harsh discipline at age 3, and cumulative family adversity by age 6 each forecast children’s bully, victim, and bully-victim statuses at age 7.5, above and beyond early behavioral problems (de Vries et al., 2018). Meta-analytic evidence further indicates that low warmth, hostility, and inconsistent discipline confer small-to-moderate risk for all bullying roles across childhood and adolescence (Lereya et al., 2013). Collectively, these findings dovetail with ecological theory: children reared in chronically stressful, unsupportive homes lack secure bases, rendering them simultaneously more prone to enact aggression and to become its targets (Repetti et al., 2002). Therefore, we propose the following hypothesis:

Four complementary theories explain why these associations arise. Attachment theory argues that insecure bonds with caregivers cultivate hostile internal working models that predispose youth to both aggression and victimization (Murphy et al., 2017). Emotional-Security Theory posits that repeated inter-parental discord undermines children’s felt safety, motivating defensive patterns—vigilance, aggression, withdrawal—that spill over into peer contexts (Davies et al., 2002). Patterson’s coercive-family-process model depicts the home as a training ground where children learn aversive interaction scripts and later enact them at school (Smith et al., 2014). Finally, General Strain Theory highlights that persistent family stressors generate negative affect that some adolescents discharge through aggressive outlets such as bullying (Hay et al., 2010).

*H1*: Higher family dysfunction will be positively associated with youth bullying involvement.

### 1.2 Parental aggravation and the family stress pathway

To elucidate how dysfunctional families propel youths toward bullying involvement, we invoke the Family Stress Model (FSM), which posits that chronic stressors inside the home undermine caregivers’ psychological well-being and parenting quality, thereby jeopardizing children’s adjustment (Conger et al., 1994). Within these stressful contexts, caregivers frequently experience parental aggravation—heightened irritability, anger, and fatigue directed toward their children, which serves as a proximal mechanism linking external family problems to child maladjustment (Zimmer-Gembeck et al., 2022).

Accumulating evidence shows that financial hardship, marital conflict, and household chaos raise caregivers’ depressive symptoms and parenting stress. Stressed parents, in turn, are more likely to employ harsh, inconsistent, or emotionally unsupportive practices, offering less warmth and monitoring while displaying greater hostility (Freisthler et al., 2022; Zhang et al., 2021). Such aggravated parenting behaviors reliably forecast children’s behavioral and emotional difficulties, including aggression, anxiety, and other conduct problems.

In the bullying domain, parental aggravation appears to be a pivotal conduit. Irritated caregivers may respond to misbehavior with shouting or punitive discipline, model aggressive problem-solving, and devote less effort to mediating sibling or peer conflicts (Shao et al., 2024; Zhang et al., 2021). Over time, these patterns can socialize children toward antisocial strategies or leave them without effective coping skills, elevating the likelihood that they will bully others, be victimized, or adopt both roles. Collectively, these findings suggest that parent-focused stress reactions constitute one plausible pathway by which a dysfunctional family climate translates into bullying involvement.

*H2*: Parental aggravation will mediate the link between family dysfunction and bullying involvement, such that family dysfunction predicts increased parental aggravation, which in turn predicts greater bullying involvement.

### 1.3 Children’s mental health as a serial mediator

Family dysfunction and parenting stress rarely affect bullying involvement in isolation; rather, their influence on children is frequently indirect, operating through youngsters’ mental health and behavioral adjustment (Fraga et al., 2021; Zhang et al., 2021). In our conceptual framework, child mental health functions as a second, downstream mediator that helps illuminate how adverse family conditions culminate in bullying. Converging evidence shows that negative parenting and chronic family stress erode children’s mental health, increasing both internalizing symptoms (e.g., anxiety, low self-esteem) and externalizing difficulties (e.g., anger, conduct problems) (Birnkammer et al., 2024; Fraga et al., 2021). For example, long-term exposure to inter-parental conflict or harsh discipline predicts emotion-regulation deficits and disruptive-behavior disorders in middle childhood.

These mental-health challenges, in turn, heighten vulnerability within the peer context. Developmental-psychopathology studies consistently demonstrate that youths with pronounced emotional or behavioral problems are over-represented among both bullies and victims (Wolke and Lereya, 2015). Impulsive, highly aggressive children—often emerging from stressful home environments—are disproportionately likely to bully peers, whereas youngsters struggling with anxiety or depression may lack assertiveness and become easy targets (Roberts et al., 2021; Wolke and Lereya, 2015). Notably, “bully-victims” frequently present the most severe psychiatric impairments, a pattern confirmed by a recent Lancet meta-analysis showing that pre-existing neurodevelopmental or psychiatric conditions more than double the odds of both perpetration and victimization (Abregú-Crespo et al., 2024).

Taken together, prior work implies a sequential process: family dysfunction elevates parental aggravation; aggravated parenting undermines child mental health; compromised mental health, in turn, increases the likelihood of bullying involvement (Birnkammer et al., 2024; Chui et al., 2020; Fraga et al., 2021). Therefore, we advance the following two hypotheses:

*H3*: Children’s mental-health difficulties are expected to mediate the association between family dysfunction and bullying involvement: higher levels of family dysfunction should correspond to poorer child mental health, and poorer mental health should, in turn, correspond to greater involvement in bullying.

*H4*: The relation between family dysfunction and bullying involvement is expected to be sequentially mediated by parental aggravation and child mental difficulties; specifically, greater family dysfunction should be linked to more pronounced parental aggravation, which is anticipated to impair child mental health, and this impaired mental health should subsequently predict increased bullying involvement.

### 1.4 Protective role of neighborhood support (community buffering)

While family dysfunction can set in motion risk processes for bullying (Baldry and Farrington, 2000; Lereya et al., 2013), the broader social context may alter these processes. Bronfenbrenner (1979)’s ecological perspective reminds us that children and families are embedded in larger communities; supportive features of the neighborhood environment can promote resilience by offsetting stress from within the home (Baldry and Farrington, 2000; Lereya et al., 2013), the broader social context may alter these processes. Bronfenbrenner (1979)’s ecological perspective reminds us that children and families are embedded in larger communities; supportive features of the neighborhood environment can promote resilience by offsetting stress from within the home (Kingsbury et al., 2020).

In this study, we focus on neighbor support, the perceived supportiveness and cohesion of the local community, as a potential moderator that buffers the impact of family stress on child outcomes (Kim et al., 2021). According to the stress-buffering hypothesis, social support can protect individuals from the deleterious effects of stress by providing emotional, informational, or instrumental resources in times of need (Cohen and Wills, 1985). For families facing high dysfunction, having trusting, helpful relationships in the neighborhood (e.g., adults who look out for each other’s children, neighbors one can turn to for advice or assistance) may alleviate some of the burden on parents and children (Kingsbury et al., 2020; Maguire-Jack and Showalter, 2016). Empirical work suggests that in cohesive, socially organized neighborhoods, parents experience lower parenting stress and children have better mental health outcomes, even under conditions of adversity.

**Figure 1:**
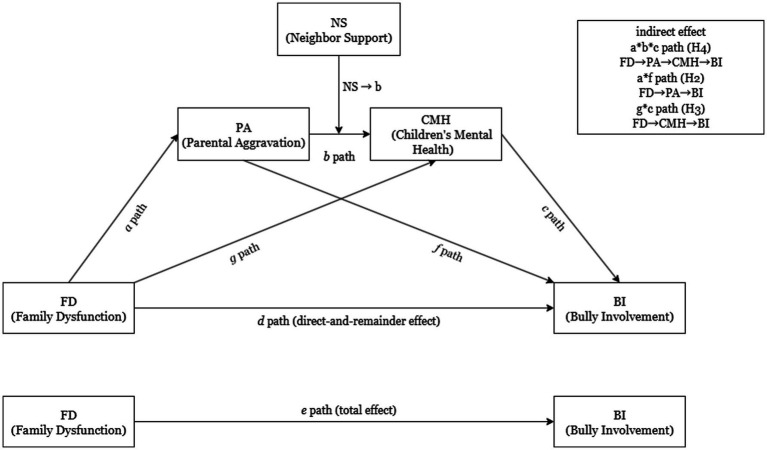
Conceptual framework of moderated mediation model.

In other words, a strong community support network can serve as a protective factor that moderates (weakens) the transmission of risk from the family to the child. We propose that neighbor support is most salient in moderating the link between parental aggravation and child mental health (Maguire-Jack and Showalter, 2016). This stage of the process, where parenting stress affects the child’s psychological well-being, could plausibly be buffered by outside support. For example, in a high-support neighborhood, an overwhelmed parent might receive encouragement or childcare help from neighbors, thereby reducing the impact of their aggravation on the child (Masarik and Conger, 2017). Likewise, a child living in a supportive community (e.g., with positive adult role models or safe peer spaces) might be less adversely affected by a parent’s irritability or stress at home (Butler et al., 2022). By contrast, in low-support neighborhoods, family stress may have a more unchecked, magnified effect on children’s mental health. Guided by this logic and prior evidence, we expect that neighbor support will moderate the indirect pathway from family dysfunction to bullying. In statistical terms, we hypothesize a moderated serial mediation, wherein the indirect effect of family dysfunction on bullying (via parental aggravation and child mental health) is attenuated under conditions of high neighbor support. Below, we articulate the specific hypotheses to be tested, integrating the above literature into a conceptual model (Figure 1) grounded in the Family Stress Model, ecological systems theory, and the stress-buffering framework.

*H5*: Neighbor support will moderate the pathway between parental aggravation and child mental health, such that this indirect effect is weaker (the impact of parenting stress on child mental health is reduced) at higher levels of community support. In other words, strong neighbor support is expected to buffer the family stress cascade, mitigating the risk of bullying involvement in high-dysfunction families.

## 2 Methods

### 2.1 Data and study population

This study draws on the 2020–2023 waves of the National Survey of Children’s Health (NSCH), a federally funded, nationally representative survey that captures the physical, emotional, and social well-being of U. S. children aged 0–17. Sponsored by the Maternal and Child Health Bureau (HRSA–MCHB) and conducted by the U. S. Census Bureau, the NSCH follows a stratified, address-based sampling design: households with children under 18 are contacted by mail, and when multiple children reside in the same household, one child is randomly selected. Across the 2020, 2021, 2022, and 2023 survey cycles, the NSCH obtained a combined total of 202,934 completed interviews, representing the non-institutionalized population of U. S. children at both national and state levels. All data-collection protocols received approval from the National Center for Health Statistics Research Ethics Review Board. Rigorous quality-control procedures—including multi-stage weighting, non-response adjustment, and data validation—ensure that the resulting dataset is both accurate and broadly generalizable.

**Figure 2:**
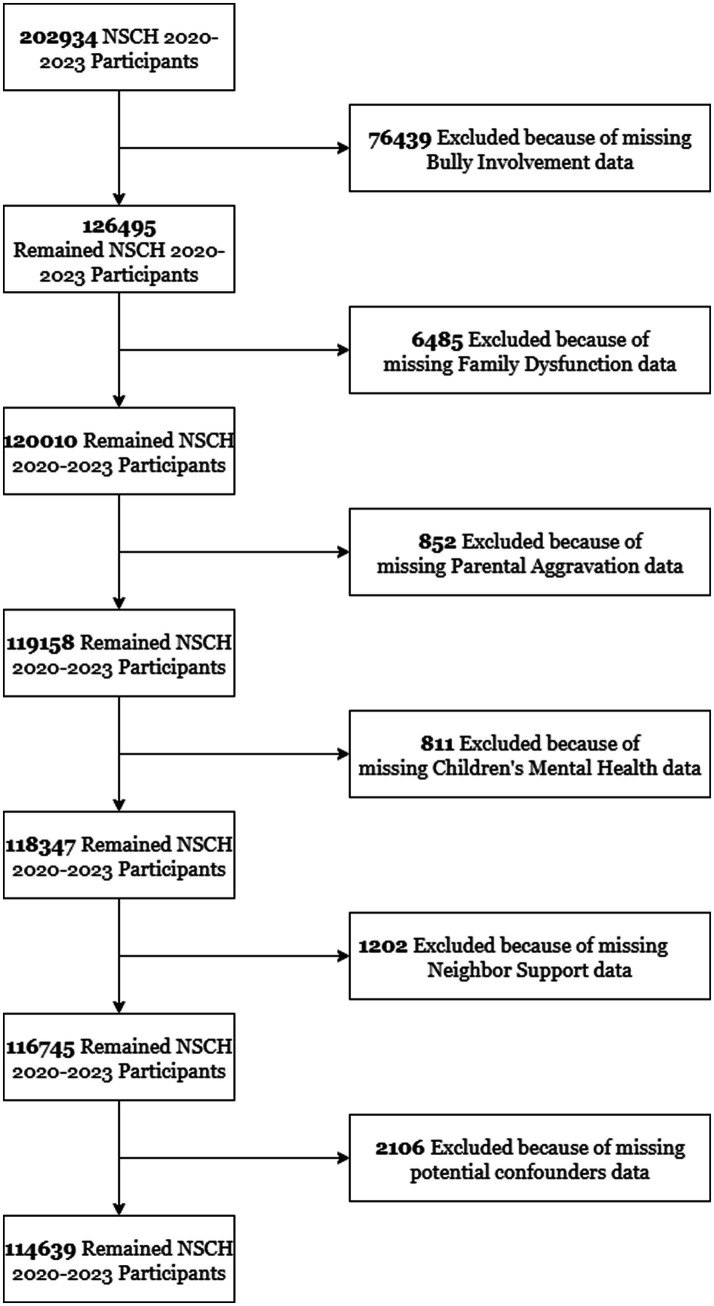
Flowchart of participant selection.

**Figure 3:**
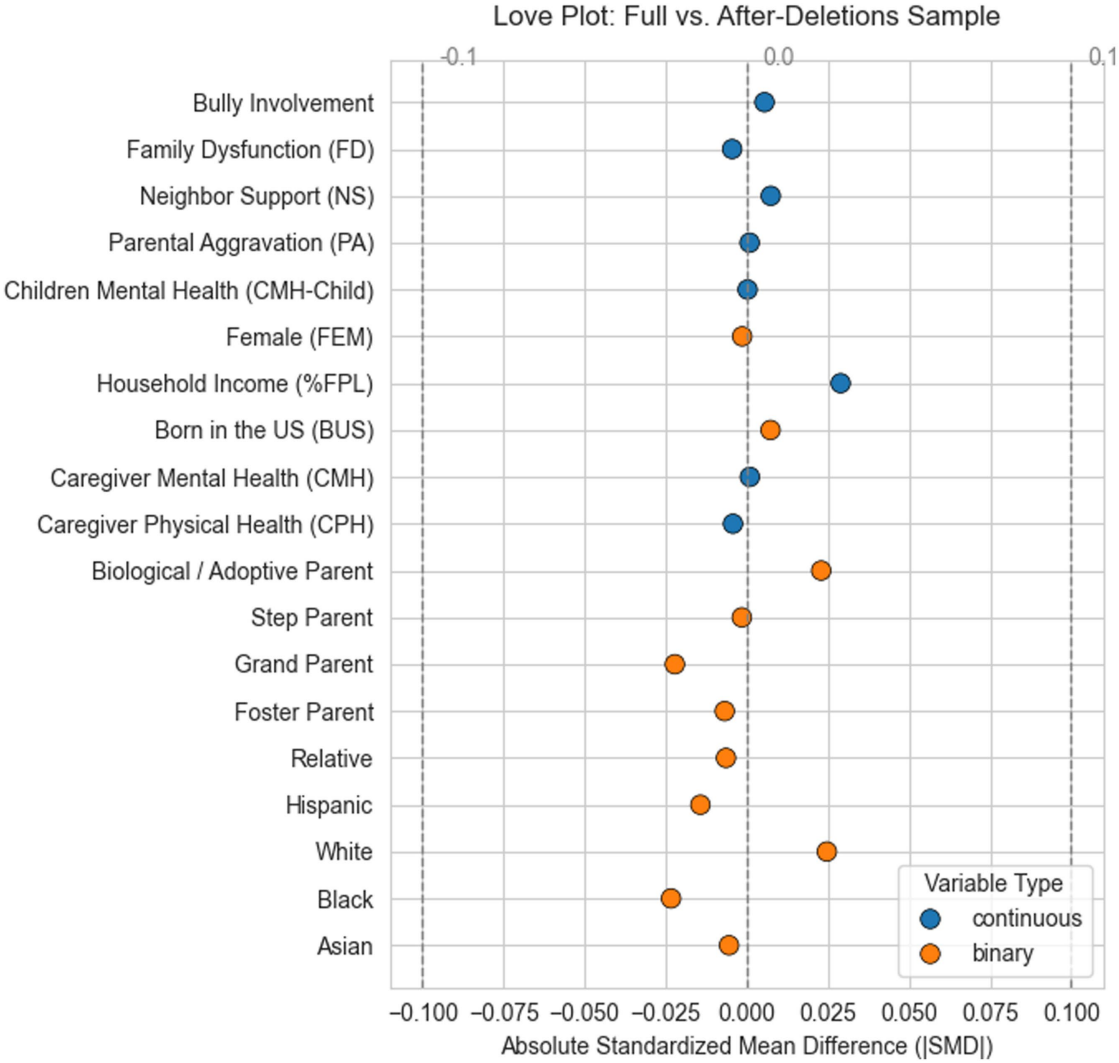
Covariate balance: Love Plot (Full vs. After-Deletions Sample).

During sample construction, we performed case-wise screening for missing data on the study’s focal variables—family dysfunction, parental aggravation, child mental health, neighborhood support, bullying involvement, and other explanatory covariates. Any record with a missing value on one or more of these indicators was removed, eliminating 88,295 observations. After cleaning, the pooled 2020–2023 NSCH files yielded a final analytic sample of 114,639 children aged 6–17 years (~52% boys, ~48% girls). The full selection procedure is depicted in Figure 2, and Figure 3 presents the love plot illustrating absolute standardized mean differences of covariates before and after exclusions.

### 2.2 Measurement

All primary variables—family dysfunction, parental aggravation, child mental health, neighborhood support, and bullying involvement—along with all study covariates were drawn from the 2020–2023 waves of the National Survey of Children’s Health (NSCH); every measure is based on parent-reported (proxy) assessments.

#### 2.2.1 Family dysfunction

The NSCH indexes family dysfunction through six household–level Adverse Childhood Experience (ACE) indicators (items 3, 4, 5, 6, 8, 9 in the 12-item ACE battery): (1): “Parent or guardian ever divorced or separated.”; (2): “Parent or guardian ever died.”; (3): “Parent or guardian ever incarcerated.”; (4): “Child ever saw or heard violence in the home.” (5): “Child ever lived with someone who had a mental-health condition.”; (6): “Child ever lived with someone who had an alcohol or drug problem.” Each item was coded 1 = Yes or 0 = No and then summed to form a composite score ranging from 0 to 6, with higher values indicating greater family dysfunction. Comparable measurement approaches have been described in prior studies (Ho et al., 2019).

#### 2.2.2 Parental aggravation

Parental aggravation was assessed in the NSCH with three items asking how often, during the past month, caregivers felt that (1) “this child is much harder to care for than most children of the same age,” (2) “this child does things that really bother you,” and (3) they felt “angry with this child.” Responses were recorded on a five-point Likert scale (never, rarely, sometimes, usually, always). All items were reverse-coded and summed so that higher scores reflect greater aggravation (possible range = 3–15). The scale showed adequate internal reliability in the present sample (Cronbach’s *α* = 0.79). Similar measurement methods are documented in earlier research (Suh and Luthar, 2020).

#### 2.2.3 Child mental health

The NSCH captures children’s internalizing-health burden with two parent-reported diagnostic items: (1): “Has a doctor or other health-care provider ever told you that this child has anxiety problems?”; (2): “Has a doctor or other health-care provider ever told you that this child has depression?.” Responses were coded 1 = Yes and 0 = No. Following Yang et al. (2023), we reverse-coded the responses so that 1 indicates the absence of the condition and 0 its presence, then summed the two items to create a composite ranging from 0 to 2. Higher scores, therefore, reflect poorer mental health.

#### 2.2.4 Neighborhood support

In the NSCH, caregivers of children aged 6–17 answered four items (I16A–I16D) tapping neighborhood social cohesion and support: (1): “People in this neighborhood help each other out.”; (2): “People in this neighborhood watch out for each other’s children.”; (3): “There are people I can count on in this neighborhood.”; (4): “If this child were outside playing and got hurt or scared, there are adults nearby whom I trust to help.” For each statement, respondents indicated whether they definitely agree, somewhat agree, somewhat disagree, or definitely disagree (coded 1–4, respectively). All items were reverse-coded so that higher values reflected greater neighborhood support and then summed to create a composite score ranging from 4 to 16. Earlier studies report the use of similar measurement strategies (Jones et al., 2024).

#### 2.2.5 Bullying involvement

In the NSCH, parent reports captured bullying involvement for children aged 6–17 through two mirror-image items. The first asked, “During the past 12 months, how often did this child bully others, pick on them, or exclude them?”; the second asked, “During the past 12 months, how often was this child bullied, picked on, or excluded by other children?” In both cases, respondents were instructed to ignore incidents involving siblings (all 6–17-year-olds) and dating partners (12–17-year-olds). Each item used an identical five-point frequency scale: 1 = Never (past 12 months), 2 = 1–2 times (past 12 months), 3 = 1–2 times per month, 4 = 1–2 times per week, and 5 = Almost every day. A composite bullying-involvement score was then created by summing the perpetration and victimization ratings, with higher totals indicating greater overall engagement in bullying.

#### 2.2.6 Explanatory covariates

Guided by the “good controls” principles articulated by Rubin (2009), Gelman et al. (2021), and Cinelli et al. (2024), we included only covariates that plausibly precede—and are therefore not influenced by—the treatment. Drawing on the dataset, we adjusted for the focal child’s sex and race, the respondent’s relationship to the child, caregiver sex, nativity (U. S.- vs. foreign-born), employment status, self-reported physical and mental health, and household income expressed as a percentage of the federal poverty level (FPL). The NSCH imputes the child’s sex, race, and household income using census-linked administrative data; full details appear in the: https://www.childhealthdata.org/learn-about-the-nsch/methods. Sensitivity analyses that sequentially exclude or add each covariate, along with alternative model specifications, yield substantively identical results. These supplementary findings are available from the corresponding author upon request.

### 2.3 Data analysis strategy

To clarify how family dysfunction shapes children’s bullying involvement, we estimated a moderated serial multiple-mediation model in which parental aggravation (M₁) and child mental health (M₂) operate as sequential mediators, and perceived neighborhood support (W) moderates the M₁ → M₂ path. All direct and indirect paths were derived with the percentile-bootstrap method and are reported as both unstandardized coefficients and percentage coefficients ( $b_{p}$ ) (Zhang et al., 2023). The $b_{p}$ metric—recommended by Zhao et al. (2025) and now common in public-health analyses (Wu et al., 2025; Zhang et al., 2025) —offers a scale-free, intuitively interpretable effect size and facilitates comparisons across paths, thereby addressing criticisms of standardized coefficients raised by King (1986), Blalock (2017), Liu and Li (2024). Indirect effects were computed as the mean product of the relevant $b_{p}$ coefficients across the bootstrap iterations, enabling formal comparisons among competing mediation chains within the same model (Zhao et al., 2025). For implementation details and replication code (see Zhao X. et al., 2024; Han et al., 2023). All the analyses were implemented in Python 3.12 and R 4.5.

## 3 Results

### 3.1 Participant characteristics

Table 1 presents the sociodemographic and psychosocial characteristics of the 114,639 children in the analytic cohort and contrasts those with and without bully involvement. The sample was evenly split by sex (female, 48.2%); most participants were born in the United States (95.9%) and identified as White (65.9%), with Hispanic (14.1%), Black (6.2%), and Asian (5.9%) children comprising the remainder.

**Table 1:** Descriptive statistics of the independent, dependent, mediating, moderating, and controlling variables (*n* = 114,639).

Variables	Overall (*N* = 114,639)	Bully involvement (*N* = 49,471)	Bully non-involvement (*N* = 65,168)	*P*-value
Parental aggravation	5.367 (0.006)	6.110 (0.010)	4.803 (0.007)	<0.001
Children’s mental health	0.258 (0.002)	0.403 (0.003)	0.148 (0.002)	<0.001
Family dysfunction	0.650 (0.003)	0.834 (0.006)	0.511 (0.004)	<0.001
Household income as a percentage of the federal poverty level	288.147 (0.367)	291.861 (0.546)	285.327 (0.495)	<0.001
Caregiver’s mental health	2.084 (0.003)	2.303 (0.004)	1.918 (0.003)	<0.001
Caregiver’s physical health	2.188 (0.003)	2.314 (0.004)	2.092 (0.003)	<0.001
Neighbor support	12.294 (0.008)	11.875 (0.012)	12.612 (0.010)	<0.001
Child gender (*n* %)				<0.001
Male	59,427 (51.8)	25,084 (50.7)	34,343 (52.7)	
Female	55,212 (48.2)	24,387 (49.3)	30,825 (47.3)	
Born in the US (*n* %)				<0.001
Yes	109,969 (95.9)	48,051 (97.1)	61,918 (95.0)	
No	4,670 (4.1)	1,420 (2.9)	3,250 (5.0)	
Relationship with selected child (*n* %)				
Biological or adoptive parent	104,606 (91.2)	44,996 (91.0)	59,610 (91.5)	0.002
Step parent	2,923 (2.5)	1,361 (2.8)	1,562 (2.4)	<0.001
Grand parent	5,253 (4.6)	2,314 (4.7)	2,939 (4.5)	0.184
Foster parent	224 (0.2)	128 (0.3)	96 (0.1)	<0.001
Relative	374 (0.3)	175 (0.4)	199 (0.3)	0.171
Child race (*n* %)				
Hispanic	16,150 (14.1)	5,927 (12.0)	10,223 (15.7)	<0.001
White	75,601 (65.9)	35,625 (72.0)	39,976 (61.3)	<0.001
Black	7,160 (6.2)	2,347 (4.7)	4,813 (7.4)	<0.001
Asian	6,793 (5.9)	1,611 (3.3)	5,182 (8.0)	<0.001

Marked differences emerged when the sample was stratified by bully involvement. Compared with the 65,168 children who reported no involvement, the 49,471 bully-involved children were exposed to substantially greater family strain, as reflected by higher parental aggravation (6.11 vs. 4.80) and family dysfunction (0.83 vs. 0.51) as well as poorer child mental health (0.403 vs. 0.148) (all *p* < 0.001). Caregivers of bully-involved children also reported worse mental health (2.30 vs. 1.92) and physical health (2.31 vs. 2.09), while perceived neighborhood support was lower (11.88 vs. 12.61) in this group (all *p* < 0.001). Although the mean household income was slightly higher among bully-involved children (291.9 vs. 285.3 percent of the poverty level), the absolute difference was modest despite statistical significance. Demographic patterns paralleled these psychosocial contrasts. Bully involvement was marginally more common among girls (49.3% vs. 47.3%, *p* < 0.001) and among children born in the United States (97.1% vs. 95.0%, *p* < 0.001). Racial and ethnic composition differed as well: White children represented a larger share of the bully-involved group than of the non-involved group (72.0% vs. 61.3%). Small but statistically significant differences were also observed in caregiver relationship categories; bully-involved children were slightly more likely to live with a step- or foster parent, though these categories accounted for fewer than 3 and 1% of children, respectively.

### 3.2 Bivariate correlations of key study variables

Zero-order Pearson correlations (see Table 2) showed that bully involvement was moderately associated with higher parental aggravation (*r* = 0.382, *p* < 0.001) and poorer child mental health (*r* = 0.288, *p* < 0.001) and was modestly related to greater family dysfunction (*r* = 0.206, *p* < 0.001). Consistent with a buffering hypothesis, neighbor support correlated inversely with bully involvement (*r* = −0.175, *p* < 0.001) and with each family-risk variable (*r* = −0.135 to −0.183, all *p* < 0.001). Parental aggravation and child mental-health difficulties were themselves positively correlated (*r* = 0.303, *p* < 0.001), suggesting a plausible mediational sequence tested in subsequent models.

**Table 2:** Zero-order Pearson correlations.

Variables	1	2	3	4	5
1. Bully involvement	—	0.206***	−0.175***	0.382***	0.288***
2. Family dysfunction	0.206***	—	−0.181***	0.162***	0.251***
3. Neighbor support	−0.175***	−0.181***	—	−0.183***	−0.135***
4. Parental aggravation	0.382***	0.162***	−0.183***	—	0.303***
5. Children’s mental health	0.288***	0.251***	−0.135***	0.303***	—

***p < 0.001.

### 3.3 Direct and mediated effects

**Figure 4:**
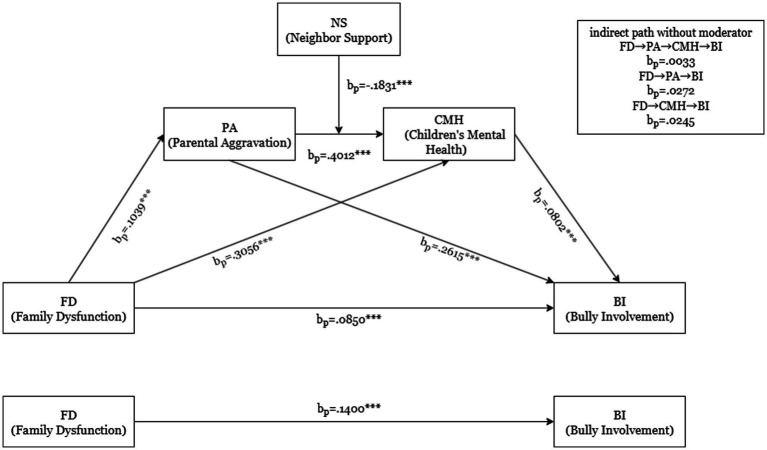
Statistical diagram of moderated mediation results.

Figure 4 and Table 3 summarize the main findings, which we discuss below based on our hypotheses. Consistent with H1, family dysfunction was positively associated with child bullying involvement. As shown in Table 3, a higher level of family dysfunction predicted greater bullying involvement in a linear regression model (*β* = 0.1133, $b_{p}$ = 0.0850, *p* < 0.001). In other words, children from more dysfunctional family environments were more likely to be involved in bullying. These results support H1.

**Table 3:** Regression analyses of mediation and moderation effects.

Right: Equation ID	Mediation analysis			Moderation analysis	Total effect analysis
	Equation I	Equation II	Equation III	Equation IV	Equation V
Right: Dependent variable (DV)	Parental aggravation	Children’s mental health	Bully involvement	Children’s mental health	Bully involvement
1. Intercept	0.1375***	−0.0771***	−0.0033	−0.0571***	0.0309***
2. Female (FEM)	−0.0207 (−0.2479)***	0.0494 (0.0989)***	−0.0000 (−0.0170)	0.0491 (0.0981)***	−0.0021 (−0.0170)*
3. Race (reference “Multiracial”)					
Asian	0.0024 (0.0293)	−0.0440 (−0.0880)***	−0.0332 (−0.2653)***	−0.0449 (−0.0898)***	−0.0360 (−0.2878)***
Black	−0.0140 (−0.1681)***	−0.0279 (−0.0558)***	−0.0145 (−0.1161)***	−0.0304 (−0.0608)***	−0.0209 (−0.1669)***
Hispanic	−0.0147 (−0.1761)***	0.0003 (0.0006)	−0.0109 (−0.0875)***	−0.0012 (−0.0024)	−0.0152 (−0.1218)***
White	−0.0077 (−0.0919)***	0.0228 (0.0456)***	0.0132 (0.1055)***	0.0249 (0.0498)***	0.0128 (0.1022)***
4. Relationship with selected child (reference “non-relative”)					
Biological or adoptive parent	−0.0194 (−0.2322)***	0.0185 (0.0371)*	0.0159 (0.1273)***	0.0189 (0.0377)*	0.0117 (0.0937)**
Step parent	0.0139 (0.1671)*	−0.0198 (−0.0396)*	0.0077 (0.0613)	−0.0182 (−0.0364)*	0.0102 (0.0814)*
Foster parent	0.1081 (1.2975)***	0.0121 (0.0241)	0.0374 (0.2989)***	0.0159 (0.0317)	0.0701 (0.5607)***
Grand parent	−0.0039 (−0.0471)	−0.0238 (−0.0476)**	0.0034 (0.0275)	−0.0210 (−0.0420)*	0.0004 (0.0030)
Relative	0.0085 (0.1019)	−0.0031 (−0.0063)	0.0115 (0.0916)	−0.0020 (−0.0041)	0.0137 (0.1096)
5. Household income as a percentage of the federal poverty level (FPL)	0.0413 (0.0014)***	0.0070 (0.0000)**	−0.0031 (−0.0001)*	0.0109 (0.0001)***	0.0096 (0.0002)***
6. Born in the US (BUS)	0.0223 (0.2672)***	−0.0002 (−0.0003)	−0.0069 (−0.0555)**	−0.0007 (−0.0015)	−0.0004 (−0.0032)
7. Caregiver’s mental health (GMH)	0.2164 (0.6493)***	0.1107 (0.0553)***	0.0529 (0.1059)***	0.1025 (0.0512)***	0.1254 (0.2507)***
8. Caregiver’s Physical health (GPH)	−0.0140 (−0.0421)***	0.0315 (0.0158)***	0.0136 (0.0272)***	0.0257 (0.0128)***	0.0120 (0.0240)***
9. Family dysfunction (FD)	0.1039 (0.2079)***	0.3056 (0.1019)***	0.0850 (0.1133)***	0.2997 (0.0999)***	0.1400 (0.1867)***
10. Parental aggravation (PA)		0.4012 (0.0669)***	0.2615 (0.1743)***	0.5398 (0.0900)***	
11. Children’s mental health (CMH)			0.0802 (0.3210)***		
12. Neighbor support (NS)				−0.0221 (0.0032)**	
13. NS × PA				−0.1831 (−0.0020)***	
Total *R*^2^	0.100	0.158	0.207	0.160	0.089

*p < 0.05, **p < 0.01, ***p < 0.001.

Unstandardized coefficients are shown first; standardized coefficients are given in parentheses. *R*^2^ = coefficient of determination.

In Table 3, family dysfunction was associated with higher parenting aggravation (*β* = 0.2079, $b_{p}$ = 0.1039, *p* < 0.001), indicating that caregivers in dysfunctional family contexts reported significantly more stress and aggravation in parenting. Second, higher parental aggravation in turn predicted greater bullying involvement when controlling for family dysfunction (*β* = 0.1743, $b_{p}$ = 0.2615, *p* < 0.001). Parents who felt more aggravated by parenting had children who were more frequently involved in bullying, even after accounting for the direct effect of family dysfunction. The indirect effect of family dysfunction on bullying involvement through parental aggravation was statistically significant. The bootstrapped indirect effect was $b_{p}$ = 0.0272 (95% CI [0.0253, 0.0291]), indicating that a substantial portion of the total effect was transmitted via parenting stress (see details in Table 4). Notably, in this single-mediator model, the direct effect of family dysfunction on bullying remained positive and significant (*p* < 0.001), suggesting partial mediation. These results are in line with H2.

**Table 4:** Indirect effect.

Indirect effect		Effect	BootSE	BootLLCI	BootULCI
Without moderator	Family dysfunction → Parental aggravation → Bully involvement	0.0272	0.0010	0.0253	0.0291
	Family dysfunction → Children’s mental health → Bully involvement	0.0245	0.0008	0.0230	0.0261
	Family dysfunction → Parental aggravation → Children’s mental health → Bully involvement	0.0033	0.0001	0.0031	0.0036
Index of moderated mediation (Family dysfunction → Parental aggravation → Children’s mental health → Bully involvement)		−0.0015	0.0003	−0.0020	−0.0010

H3 proposed that child mental health problems mediate the association between family dysfunction and bullying involvement. The results supported this hypothesis as well. Family dysfunction was a strong predictor of child mental health difficulties: children from more dysfunctional families exhibited significantly worse mental health (*β* = 0.1019, $b_{p}$ = 0.3056, *p* < 0.001). In turn, poorer child mental health was associated with greater bullying involvement, controlling for family dysfunction (*β* = 0.3210, $b_{p}$ = 0.0802, *p* < 0.001). That is, children with more mental health problems were more likely to bully others or be victimized by bullying. In Table 4, the indirect effect of family dysfunction on bullying through child mental health was significant, with a bootstrapped $b_{p}$ = 0.0245 (95% CI [0.0230, 0.0261]). As with H2, this mediation was partial: the direct effect of family dysfunction on bullying remained significant in this single-mediator model (*p* < 0.001), though its magnitude was reduced. These findings support H3 by identifying child mental health as an important mediating pathway.

H4 posited a sequential mediation, whereby family dysfunction leads to increased parenting stress, which in turn leads to worse child mental health, ultimately resulting in greater bullying involvement. We tested a path model including both parental aggravation and child mental health as mediators in sequence (see Table 3). All path coefficients in the sequence were significant. As noted, family dysfunction predicted higher parental aggravation (β = 0.2079, $b_{p}$ = 0.1039, *p* < 0.001), and higher parental aggravation was associated with more child mental health problems (*β* = 0.0669, $b_{p}$ = 0.4012, *p* < 0.001). In turn, elevated child mental health problems predicted greater bullying involvement (*β* = 0.3210, $b_{p}$ = 0.0802, *p* < 0.001). Taken together, the results indicate that family dysfunction increases parenting stress, which in turn erodes child mental health, ultimately elevating the risk of bullying involvement. This finding supports H4’s proposed sequential mediation.

### 3.4 Moderated mediation

H5 predicted that perceived neighborhood support would moderate the stress cascade, buffering the effect of parenting stress on child mental health (and thereby weakening the overall indirect path from family dysfunction to bullying). The findings provided clear support for this moderated mediation hypothesis. As shown in Table 3, there was a significant interaction between parental aggravation and neighborhood support in predicting child mental health problems (*β* = −0.0020, $b_{p}$ = − 0.1831, *p* < 0.001). The negative coefficient for the interaction term indicates that the detrimental impact of parenting aggravation on children’s mental health was attenuated at higher levels of neighborhood support. In other words, in contexts where neighbors were perceived as supportive, helpful, and trustworthy, high parenting stress was less likely to translate into severe mental health difficulties for the child. Conversely, when neighborhood support was low, the association between parental aggravation and child mental health was much stronger — implying that children in unsupportive neighborhoods bore the full brunt of their parents’ stress. Figure 4 illustrates this interaction effect: the slope linking parenting aggravation to child mental health is steep under low-support conditions but relatively flat when support is high.

Because of this buffering effect, the indirect pathways from family dysfunction to bullying became conditional on neighborhood support. In particular, the strength of the serial mediation varied significantly by support level (Table 4). When neighborhood support was entered as a moderator, the sequential indirect path from family dysfunction to bullying involvement (via parental aggravation and children’s mental health) was significant but very small ( $b_{p}$ = − 0.0015, 95% CI [−0.0020, −0.0010]). In practical terms, this means that in poorly supported neighborhoods, a dysfunctional family environment was far more likely to set off a chain reaction of stressed parenting and child mental-health problems culminating in bullying involvement. In well-supported neighborhoods, that harmful chain was much weaker. Strong community cohesion helped to short-circuit the process linking family stress to bullying. Stated differently, children living in highly supportive neighborhoods were less affected by family dysfunction and parental stress when it came to their mental health and bullying outcomes, compared to children in low-support neighborhoods. This moderated mediation pattern confirms H5: neighbor support buffered the family stress–bullying link, significantly reducing the indirect risk pathway.

## 4 Discussion

In line with extensive evidence linking adverse family conditions to youth bullying involvement, we found that children from more dysfunctional families had higher odds of bullying involvement (Bowes et al., 2009; Fraga et al., 2021). Family dysfunction increased parenting stress, which in turn compromised children’s mental health, ultimately elevating their risk of engaging in or suffering from bullying (Duke et al., 2010; Fraga et al., 2021).

### 4.1 Theoretical implications in an ecological context

Family dysfunction, which includes conflict, lack of cohesion, or other adversities, appears to initiate a chain reaction: it heightens caregivers’ aggravation and stress in the parenting role, likely undermining the emotional support and guidance they provide. This, in turn, is associated with children’s worsened mental health (e.g., greater anxiety, depression, or behavioral dysregulation), explaining why these youth are more prone to bullying others. This dynamic is consistent with the Family Stress Model, wherein chronic family stressors disrupt parenting and thereby increase the risk of child behavior problems (Conger et al., 1994; Masarik and Conger, 2017; Repetti et al., 2002). Moreover, our results align with the stress-buffering hypothesis: even under severe family strain, a cohesive neighborhood can help interrupt the progression from parental stress to child maladjustment.

Bronfenbrenner’s ecological systems theory emphasizes that a child’s development is embedded in nested contexts ranging from immediate family to broader community (Bronfenbrenner, 1977). Our moderated mediation results empirically illustrate this multi-level interplay. Consistent with the classic stress-buffering hypothesis, high neighborhood support attenuated the deleterious impact of parental aggravation on children’s mental health. This finding underscores that family processes do not operate in isolation—broader contextual factors can fundamentally alter how family risk translates into youth behavioral outcomes (Nomaguchi and Fettro, 2020; Zhang et al., 2021). This finding is theoretically important because it bridges micro-level and meso/ecosystem-level frameworks, suggesting a more integrative model of bullying etiology. In essence, the study suggests that while dysfunctional family dynamics and parenting stress create vulnerability for youth aggression, the presence of strong social support in the surrounding community can intercept this vulnerability before it manifests as bullying (Strøm et al., 2014). It also extends prior work on family resilience and youth behavior by identifying neighborhood support as a contextual resilience factor that deserves attention.

Serial mediation as an interlocking mechanism. Beyond showing that family dysfunction, parenting stress and child mental health each matter, our data illuminate how these elements interlock. The sequential pathway—family dysfunction → parental aggravation → child mental-health difficulties → bullying involvement—was statistically robust ( $b_{p}$ = 0.0033, 95% CI [0.0031, 0.0036]) even after extensive covariate control. Although the effect size is small, its presence at population scale underscores a cascading process that moves from the microsystem (family stress) through the parent–child relationship (aggravation) into the child’s internal milieu (mental health) before surfacing in the peer ecology as bullying. This pattern dovetails with family-stress and developmental-cascade models, suggesting that interventions aimed solely at the child or the parent may be insufficient: dismantling the chain requires simultaneous support for family functioning, parent emotion regulation and child psychological well-being. In short, the serial mediation highlights a narrow but critical conduit through which household adversity becomes social harm—precisely the kind of multi-level mechanism that demands coordinated, rather than siloed, preventive action.

### 4.2 Buffering role of neighborhood support

One of the most noteworthy implications of this research is the buffering effect of neighborhood support on the pathway from parenting stress to child mental health. Practically, such conditions can alleviate parents’ burden and distress: a parent who feels supported by those around them may be better able to cope with the challenges of parenting in a dysfunctional family situation, thereby reducing the intensity of negative emotions (aggravation) transmitted to the child. Moreover, even if a parent is highly stressed or aggravated, a supportive neighborhood could provide alternative resources for the child, such as other trusted adults to confide in, safe spaces to play and socialize, and positive role models. These community-provided resources likely help shore up the child’s mental health, counteracting some of the harm that might otherwise result from an overburdened or irritable parent. Our findings empirically back this interpretation: when neighborhood support was high, the correlation between parental aggravation and poor child mental health was significantly weaker. Notably, we conceptualized neighborhood support as the perceived mutual aid, trust, and cohesion among neighbors-essentially an interpersonal component of community social capital (Bowes et al., 2009; Sampson et al., 1997; Ungar, 2011). Focusing on this facet of social capital highlights the unique protective role of neighborly support in buffering children against family stress. For instance, prior research found that neighborhood social capital moderated the association between maternal depression and adolescent behavior problems, effectively dampening the negative effects of maternal mental health issues on youth outcomes (Lew et al., 2022; Zhao Y. J. et al., 2024). Similarly, our study suggests that strengthening community bonds and supports can be a crucial strategy for enhancing child and family resilience.

It is important to note that neighborhood support specifically moderated the connection between parenting stress and child mental health, rather than the initial impact of family dysfunction on parenting stress. This pattern suggests that even in dysfunctional families where high stress is perhaps inevitable, the community’s role becomes critical at the point where the child would otherwise internalize the fallout of that stress. In high-support neighborhoods, parents reporting feelings of aggravation did not have children with as poor mental health outcomes as would be expected in low-support neighborhoods (Rytilä-Manninen et al., 2014). This points to a specific leverage point for buffering interventions: enhancing neighborhood or community support may fortify children’s well-being despite parenting challenges. Such conditional mediation reflects a nuanced reality that purely individual- or family-level models might miss-namely, that context can alter the child’s vulnerability to family risk factors.

### 4.3 Implications for practice

Addressing family dysfunction and associated stressors should be the first line of defense (Hogan et al., 2002; MacPhee et al., 2015; Masarik and Conger, 2017). Briefly, evidence-based parenting programs that teach frustration tolerance, mindful discipline, and conflict-resolution skills can dampen caregivers’ aggravation and prevent its spill-over to children. Where parents report depression or chronic stress, prompt referral to counseling or peer-led support groups is essential; improving parental wellbeing often improves the whole home climate (Lancaster et al., 2023). These family-centered services address the root conditions that precipitate children’s behavioral and emotional problems, thereby shrinking the breeding ground for bullying (Hogan et al., 2002).

Schools and pediatric providers should screen routinely for mental-health symptoms in pupils exposed to family adversity or high parent stress. Early, low-intensity interventions—cognitive-behavioral groups, social–emotional learning curricula, or mentoring schemes—can strengthen emotion regulation and coping, lowering the odds that distress is expressed as aggression. Anti-bullying curricula should embed empathy training and conflict-management drills, and schools should invite parents to parallel workshops so that home and school messages reinforce each other (Kafel, 2020).

Our findings give warranted prominence to community cohesion as a buffer. Local community organizations and leaders can facilitate informal support networks among families – for example, regular meet-ups, mutual aid groups, or neighborhood watch programs – to build trust and mutual assistance. By nurturing such neighborly cohesion, communities create a collective safety net that supports struggling parents and protects vulnerable children (Bowes et al., 2009; Cohen and Wills, 1985; MacPhee et al., 2015). In short, our moderated mediation model points to a multilevel prevention agenda. By pairing parent support with child skills training and robust community ties, practitioners and policymakers can jointly disable the pathways that link household adversity to peer aggression.

### 4.4 Limitations and future directions

The present findings should be interpreted in light of several limitations. Most critically, our cross-sectional design precludes causal inference; bullying may escalate family stress as readily as family stress fuels bullying, and only prospective or experimental work can adjudicate direction (Bollen and Pearl, 2013; Zhao et al., 2010). All focal constructs were reported by the same caregiver at one time point, raising shared-method bias and the possibility of under-reporting children’s bullying due to fear of social censure. Future studies would benefit from multi-informant designs that blend parent, child, teacher, and peer data. Brief indices also constrained nuance: the six-item family-dysfunction composite collapsed heterogeneous adversities, and child mental health measures focused on parent-reported anxiety or depression, omitting other internalizing and externalizing problems pertinent to aggression. Furthermore, nearly 40% of the original NSCH cohort was lost to item non-response, and retained families were somewhat more affluent and cohesive than those excluded, raising concerns about selection bias (Nonresponse in the National Survey of Children’s Health 2007, 2012). Generalizability is also limited to U. S. cultural and policy contexts, as neighborhood cohesion may operate differently in societies with stronger communal norms or different welfare infrastructures (Greenland et al., 2016; Li et al., 2025). Finally, neighborhood support was measured perceptually; objective markers such as crime rates, collective-efficacy audits, or geocoded resources could clarify whether it is subjective trust, concrete amenities, or both that buffer family stress.

Future research should adopt longitudinal, multi-wave designs to examine reciprocal dynamics over time (Zhang et al., 2024); disaggregate specific forms of family adversity and expand assessments to encompass a broader range of child psychopathology; incorporate data from teachers, peers, and administrative records to reduce mono-informant bias; and replicate the moderated serial mediation model across diverse cultural contexts using objective indicators of neighborhood support. By addressing these limitations, research can more conclusively test the complex pathways linking family stress, neighborhood cohesion, child mental health, and bullying behaviors.

## 5 Conclusion

In conclusion, this study demonstrates a complex yet coherent pathway by which family and community contexts jointly influence a child’s risk of involvement in bullying. The findings indicate that children who grow up in dysfunctional family environments are more likely to experience stressed and aggravated parenting, which can undermine their mental health; these children, in turn, are at heightened risk for becoming involved in bullying as victims or perpetrators. Importantly, however, the presence of a supportive, cohesive neighborhood can act as a protective shield, weakening the adverse chain reaction that leads from troubled families to bullying behavior. These results underscore the notion that bullying is not merely an isolated behavior problem but rather a symptom of broader contextual challenges. Efforts to reduce bullying and improve youth outcomes should therefore extend beyond the schoolyard, reaching into the family home and the neighborhood. By addressing family dysfunction (through supporting families in crisis), alleviating parenting stress (through parent-focused interventions), and promoting child mental health (through early identification and treatment), we can tackle the root factors that feed into bullying. Furthermore, by strengthening neighborhood support networks and community engagement, society can provide vulnerable children and parents with additional buffers against adversity. By fostering healthier family dynamics and more supportive communities, we can create an environment in which children are less likely to engage in or suffer from bullying, thereby promoting safer and more nurturing developmental trajectories for all youth.

## Data Availability

Publicly available datasets were analyzed in this study. This data can be found: https://www.childhealthdata.org/learn-about-the-nsch/topics_questions/2022-nsch-guide-to-topics-and-questions/.
